# Editorial: Cellular transport and metabolism of nutrients, natural toxins, pollutants, and drugs in the digestive system of fish and aquatic invertebrates

**DOI:** 10.3389/fphys.2022.1039186

**Published:** 2022-10-03

**Authors:** Carlos M. Luquet, Flavia Bieczynski, Carol Bucking

**Affiliations:** ^1^ Subsede INIBIOMA-CEAN Laboratory of Aquatic Ecotoxicology, Consejo Nacional de Investigaciones Científicas y Técnicas (CONICET), Buenos Aires, Argentina; ^2^ Consejo Nacional de Investigaciones Científicas y Técnicas (CONICET), Buenos Aires, Argentina; ^3^ Department of Biology, York University, Toronto, ON, Canada

**Keywords:** membrane transport, intracellular effects, enterocytes, biotransformation, detoxification pathways

This Research Topic covers integrative molecular and physiological approaches focusing on intestinal alterations (caused by hormones or food contaminants) that induce or impair fish growth conditions (Meirelles et al.; Barany et al.). In addition, two review papers (Romersi and Nicklisch; Bieczynski et al.) summarize our current understanding of membrane transporters localized in the digestive system of fish and other aquatic animals as they transport nutrients and xenobiotics.

Specifically, Meirelles et al. analyzed the growth performance of transgenic zebrafish overexpressing growth hormone (GH) together with physiological, morphometric, and molecular effects caused by the expected increase in GH secretion. Effectively, GH overexpression increases zebrafish growth variables and feed intake (hyperphagia). Furthermore, these effects are associated with morphometric changes in the intestine including: increased intestinal length and mass, enterocyte height, and longer microvilli; which result in an enlargement of the intestinal surface area for absorption. In addition, they report the induction of the mRNA expression of the crucial dietary peptide transporter PEPT1 (slc15a1). Finally, the authors also show the involvement of GH in the observed changes through the induction of the GH receptor ghrb and the insulin-like growth factor 1a (igf1a) in the intestine. The authors conclude that GH overexpression increases the expression of its receptor, with the consequent induction of igf1a, leading to increased intestinal absorption area through morphometric changes along with an increase in the expression of membrane transporters needed to efficiently uptake nutrients and thus support increased body growth.

Secondly, Barany et al. explored the effects of the mycotoxin aflatoxin B1 (AFB1) on the gastrointestinal tract of the seabream (*Sparus aurata*). The authors theorized that this toxin was the cause of the growth impairment that was previously observed and sought to determine the mechanism of action. The study used both *in vitro* and *in vivo* exposure to realistic AFB1 concentrations (8 µM and 16 µM) to evaluate the integrity and function of the intestinal mucosa. Electrophysiological measurements of transepithelial resistance (TER) and short-circuit currents (Isc), histopathological analysis, and mRNA expression of tight junction-related proteins (claudins and occludins) were conducted to provide an integrative picture of intestinal function in response to this toxin. The authors found no short-term effects of AFB1on the intestinal barrier function. In contrast, long-term exposure to AFB1 caused TER changes with histopathological damage and changes in the mRNA expression of both claudins and occludins. They concluded that these changes to intestinal function likely contributed to growth impairment, as described in previous studies.

Finally, two review papers, Romersi and Nicklisch; Bieczynski et al., present an overview of the current knowledge on the gastrointestinal ATP Binding Cassette and Solute Carrier proteins (ABC and SLC) of aquatic species. These proteins are involved in the transport of ions, physiologic metabolites and xenobiotics, and nutrient absorption. Members of both protein groups play an essential role in a highly conserved defense mechanism known as multidrug resistance (MDR), also described for aquatic species as multixenobiotic resistance (MXR).

Especially, Romersi and Nicklisch review the under-researched SLC proteins that include numerous nutrient, ion, and xenobiotic transporters. They summarize the interaction of xenobiotics and physiological compounds with these proteins at gene expression and functional levels and review the knowledge on the regulation of these proteins by diet and environmental stressors. Finally, they call attention to the presence of environmental chemicals acting as competitive substrates or inhibitors of MXR and nutrient uptake transporters (Transporter-Interfering Chemicals, TICs) in the prey species of commercial fish and in the components of aquafeeds to produce healthy fishery and aquaculture products.

Further, Bieczynski et al. review the current information on all ABC subfamilies’ identification and characterization and focus on their localization and physiological and toxicological role in the fish intestine. The authors highlight the intestine’s barrier functions and its epithelium polarity, paying particular attention to the most extensively studied fish ABC proteins (subfamilies ABCB, ABCC, and ABCG), their apical or basolateral location, distribution along the intestine, and regulatory mechanisms. The authors conclude that fish intestinal ABCB, ABCC, and ABCG proteins are essential in transporting physiological substrates and aquatic pollutants, such as pesticides, cyanotoxins, metals, hydrocarbons, and pharmaceutical products. Still, much work is needed to obtain a complete picture of fish ABC proteins’ physiological and toxicological functions.

